# Information needs during an emerging outbreak of meningococcal W135 disease in the Netherlands: a study among teenagers, their parents and healthcare professionals

**DOI:** 10.1186/s12889-021-11228-3

**Published:** 2021-08-12

**Authors:** Marion de Vries, Feray R. Çoban, Liesbeth Claassen, Margreet J. M. te Wierik, Danielle R. M. Timmermans, Aura Timen

**Affiliations:** 1 National Institute for Public Health and the Environment (RIVM), Center for Infectious Disease Control, Bilthoven, Netherlands; 2 National Institute for Public Health and the Environment (RIVM), Center for Infectious Disease Control / Center for Environmental Safety and Security, Bilthoven, Netherlands; 3 Department of Public and Occupational Health, Amsterdam UMC, Vrije Universiteit Amsterdam, Amsterdam, Netherlands; 4 VU University Amsterdam, Athena Institute for Research on Innovation and Communication in Health and Life Sciences, Amsterdam, Netherlands

**Keywords:** Meningococcal disease, Vaccination, menACWY, Information needs, Adolescents, Parents, Healthcare professionals, General practitioners, Communication, Outbreak

## Abstract

**Background:**

To counter the rise in invasive meningococcal disease (IMD) serogroup W, the Netherlands introduced a menACWY catch-up vaccination campaign for teenagers in 2018 and 2019. Teenagers and parents who have questions or who seek advice from a professional about vaccinations are likely to consult a youth healthcare professional or their general practitioner. This study aimed to appraise the ability of these healthcare professionals to meet the information needs of teenagers and their parents at the start of the vaccination campaign.

**Methods:**

With online surveys, we assessed information needs in teenagers (*N* = 1603) and parents (*N* = 1784) concerning IMD and the menACWY vaccination, and in healthcare professionals (*N* = 520) in their communication with teenagers and parents. We additionally studied healthcare professionals’ expectations of the information needs of teenagers and parents.

**Results:**

We identified several information needs about IMD and the menACWY vaccination in teenagers, parents and healthcare professionals. Some important commonalities in the information needs in these three groups were found, with regard to the topics IMD prevention, vaccine effectiveness and vaccine protection duration. Healthcare professionals’ expectations of the information needs of teenagers and parents were quite accurate but some important discrepancies were found.

**Conclusion:**

Our results suggest that healthcare professionals might not have been optimally equipped or prepared for questions from teenagers and their parents at the beginning of the vaccination campaign. We recommend public health institutes to timely assess and meet information needs about new vaccines in healthcare professionals to optimally equip them for consultations.

## Background

Between 2015 and 2018, there has been a rapid increase in the number of cases with invasive meningococcal disease (IMD) caused by *Neisseria meningitidis* strain W135 in the Netherlands. The prevalence rose from an annual average of 4 cases before 2015 to 103 in 2018 [1]. Individuals from all age groups were at risk but the disease was mostly seen among children under the age of three, adolescents and elderly. The average case fatality rate (CFR) of IMD W between 2015 and 2019 was 16%. The CFR was highest among adolescents aged 14–24 years old with 26% [1].

In response to the rise in IMD W cases, the Ministry of Health, Welfare and Sports decided to replace the meningococcal C (MenC) vaccination in the National Immunization Program (NIP) with the menACWY conjugate vaccine for children aged 14 months from May 2018 onwards. In addition, teenagers aged 14–18 years were invited for a menACWY catch-up campaign in 2018 and 2019 [1].

The National Institute for Public Health and the Environment (RIVM) coordinates the NIP and catch-up vaccinations in the Netherlands. In this role, the RIVM also develops and distributes communication materials, such as flyers and online materials, to both the public and healthcare professionals. If members of the public have questions or seek additional advice from a professional about vaccinations, they are likely to consult a youth healthcare professional, who are involved in the implementation of the NIP and catch-up vaccinations, or their general practitioner (GP) [2]. The ability of these healthcare professionals to meet the information needs of teenagers and parents about vaccinations is important for the success of vaccination campaigns. A previous study found that limited knowledge about the disease and vaccine was an important barrier for adolescents in the UK for receiving the menACWY vaccination, whereas a recommendation of a healthcare professional was found to be an important facilitator [3]. Other studies showed that healthcare workers were more likely to recommend a vaccine to their client if they themselves were knowledgeable about the disease and the specific vaccine [4], and if they felt comfortable explaining the risks and benefits of the vaccination [5].

Insights into the ability of healthcare professionals to meet the information needs of teenagers and parents about recommended vaccinations is therefore important in the preparation for future vaccination campaigns. Such insights are especially important during vaccination campaigns that are rapidly implemented to control an unexpected disease outbreak. During these situations, uncertainty can be high among the groups invited for the vaccination, and public health institutes and healthcare professionals face limited time to prepare communication materials and consults.

The current study aims to appraise the ability of healthcare professionals, specifically youth healthcare professionals and GPs, to meet the information needs of parents and teenagers about the rise in IMD infections and the menACWY vaccination at the start of the catch-up menACWY vaccination campaign in 2018. We studied information needs in teenagers and parents concerning IMD and the menACWY vaccination, and we studied to what extent healthcare professionals expected these information needs in teenagers and parents. In addition, we studied information needs of healthcare professionals themselves with regard to their communication with teenagers and parents about IMD and the menACWY vaccination. We asked the following research questions:

1. Which information needs concerning IMD and the menACWY vaccination did teenagers invited for the menACWY vaccination and their parents have at the start of the catch-up vaccination campaign?
2. To what extent did healthcare professionals expect the information needs that teenagers and parents had concerning IMD and the menACWY vaccination?
3. Which information needs concerning IMD and the menACWY vaccination did healthcare professionals have with regard to their communication with parents and teenagers?

## Methods

### Procedures and study population

Surveys were conducted among teenagers, parents, GPs, and youth healthcare professionals (specifically: youth healthcare physicians and youth healthcare nurses involved in the implementation of the menACWY vaccination campaign for teenagers). All surveys were in Dutch.

Between 13 and 26 September 2018, approximately two weeks prior to the first menACWY vaccination round, 3036 teenagers who were invited for this vaccination and 3002 parents of teenagers were invited for an online survey. The surveys among teenagers and parents were conducted via an online survey panel (Kantar Public, https://www.nipo.nl/panel). A more detailed description on this recruitment has been described elsewhere [6].

GPs received an invitation for an online survey via e-mail on 13 September and again on 20 September 2018 (reminder). We obtained name and address data of individual GPs in the Netherlands from a Dutch healthcare billing organization Vektis. After removing duplicates and excluding delivery failures and irrelevant email addresses, the survey was sent to 3694 email addresses.

In addition, youth healthcare professionals were invited for survey participation through the NIP newsletter from the RIVM sent on 10 September and on 11 October 2018 (reminder). The newsletter was sent to 2696 subscribers of the newsletter (mostly healthcare professionals working in youth healthcare). Both GPs and youth healthcare professionals could fill in the online survey up to 23 October 2018.

Participation to the interviews and surveys was voluntary and all respondents were informed about the general purpose of the study prior to participation. All respondents provided written informed consent for participation to the interviews and surveys. Parents or legal guardians additionally provided written informed consent for participation of respondents under the age of 16. The Clinical Expertise Center RIVM has reviewed the study protocol and concluded that this research was not subject to the Dutch law for medical research involving human subjects [7]. Our study was, therefore, exempted from seeking further ethical approval.

### Survey development

The survey questions addressed information needs regarding IMD and the menACWY vaccination and were based on results from semi-structured interviews with teenagers (*N* = 12), their parents (*N* = 10), youth healthcare professionals (*N* = 12), and GPs (*N* = 3), conducted between April and June 2018. These interviews explored knowledge, beliefs and information needs regarding IMD and the menACWY vaccination within each group [6]. The self-reported information needs and knowledge gaps identified in the interviews were translated into information items about IMD and the menACWY vaccination that were shown to the respondents of the surveys. One survey was developed based upon the interview results from both teenagers and parents, and another survey was developed based upon the interview results from youth healthcare professionals and GPs. See Table 1 for the information items shown in the surveys.

**Table 1:** All information items as provided in the surveys; the numbers in this table correspond to the numbers provided for each item in Figs. 1-3

**Item number**	**Questions and items about meningococcal disease (respondents: parents and teenagers)**	**Item number**	**Questions and items about the menACWY vaccination (respondents: parents and teenagers)**
	What would you like to receive information about? Indicate how important or unimportant the following topics about meningococcal disease are for you.* *Information about …*		What would you like to receive information about? Indicate how important or unimportant the following topics about the vaccination against meningococcal disease are for you.* *Information about …*
I1	…how you can get infected with IMD	V1	…how this vaccination protects you against IMD
I2	…what IMD exactly is	V2	…how long this vaccination protects you against IMD
I3	…how often IMD occurs in the Netherlands	V3	… to what extent this vaccination protects you against IMD
I4	…how many people die due to IMD in the Netherlands	V4	…what are the possible side-effects of this vaccination
I5	…how you can prevent IMD	V5	…what exactly the vaccine is composed of
I6	…who are most at risk for IMD	V6	…how often you need to get vaccinated against IMD
I7	…why some people are more at risk for IMD than others	V7	…how much this vaccination hurts
		V8	…where on the body this vaccination will be administered
		V9	…the research that has been conducted on this vaccination
**Item number**	**Questions and items about meningococcal disease (respondents: healthcare professionals)**	**Item number**	**Questions and items about the menACWY vaccination (respondents: healthcare professionals)**
	*Below are some topics for information about meningococcal disease.* - Indicate how (un) important the information is for you, in your communication with teenagers and their parents* - And whether or not you are familiar with this information** *Information about …*		*Below are some topics for information about the vaccination against meningococcal disease.* - Indicate how (un) important the information is for you, in your communication with teenagers and their parents* - And whether or not you are familiar with this information** *Information about …*
I1	…who the risk groups of IMD are	V1	…why the vaccination is being implemented now
I2	…how I can recognize IMD	V2	…why the vaccination is provided for this target group
I3	…how contagious IMD is	V3	…why the vaccination is not provided to other groups
I4	…how often does IMD occur	V4	…how effective the vaccine is
I5	…how to treat IMD	V5	…how long the vaccine protects
I6	…how to prevent IMD (besides vaccinations)	V6	…how many doses each child should receive
I7	…the clinical disease course of IMD W	V7	…how many people need to be vaccinated to prevent one case
I8	…asymptomatic carriage	V8	…what is known about the safety of the vaccine
		V9	…what is known about the side-effects of the vaccine
		V10	… what exactly the vaccine is composed of
		V11	…why the vaccine also contains types A and Y
		V12	…why the vaccination does not target IMD B
		V13	…what the difference is between menC vaccination and menACWY vaccination
		V14	…what the effect of vaccinating these target groups has on groups immunity

^*^ Answer categories = 1: important, 2: a bit important, 3: not (so) important

^**^ Answer categories = 1: familiar, 2: not (so) familiar

### Survey questions

The survey question that addressed information needs in teenagers and parents was: “*What would you like to receive information about? Indicate how important or unimportant the following topics about meningococcal disease/the vaccination against meningococcal disease are for you.”* (with the answer categories: not (so) important / a bit important / important). This question was followed by 7 information items about IMD and 9 information items about the menACWY vaccination (see Table 1).

Healthcare professionals were shown the same information items that were shown to teenagers and parents and were asked, separately for teenagers and for parents: *“For this question we want to ask you to put yourself into the shoes of the (parents of) teenagers who have received an invitation for the vaccination against meningococcal disease. Indicate how (un)important (parents of) teenagers would perceive the following information.”* (answer categories: not (so) important / a bit important / important).

Lastly, we explored which information needs healthcare professionals experienced themselves in their communication with teenagers and parents. The healthcare professionals were asked: “*Below are some topics for information about meningococcal disease / the vaccination against meningococcal disease. Indicate how (un)important the information is for you, in your communication with teenagers and their parents”* (answer categories: not (so) important / a bit important / important). *“And whether or not you are familiar with this information”* (answer categories: I am familiar with this information / I not (so) familiar with this information). Followed by 8 information items about IMD and 14 information items about the menACWY vaccination (see Table 1). By studying both whether healthcare professionals considered information as important in their communication with teenagers and parents and whether they were already known with this information, more specific insights could be gained about the healthcare professionals’ preparedness for consults.

### Statistical analysis

Descriptive analyses were performed for each variable in teenagers, parents and healthcare professionals. Response proportions in teenagers and parents were compared with Chi^2^ tests. Additional Chi^2^ tests were performed to compare the information needs in teenagers and parents with the expectations of these information needs by healthcare professionals. Prior to the analyses, the answer categories “important” “a bit important” and “not (so) important” were merged into two categories: “important” and “less important” (including the original categories “not (so) important” and “a bit important”). We opted to merge these response categories due to small number of responses in the “not (so) important” category.

## Results

### The study population

In total, 1603 teenagers (response rate 53%), 1784 parents (response rate 57%), 478 GPs (response rate ~ 13%^3^) and 42 youth healthcare professionals (out of 2696 subscribers of the newsletter) filled in a survey.

A description of the study samples is shown in Table 2 (for teenagers and parents) and Table 3 (for healthcare professionals).

**Table 2:** Characteristics of teenagers and parents in this study

Teenagers		Parents	
*Female – no. (%)*	810 (50.5)	*Female – no. (%)*	991 (55.5)
*Age in years: no. (%)*		*Age in years: range (mean)*	31–73 (46.5)
*12*	111 (6.9)		
*13*	611 (28.1)		
*14*	379 (23.6)		
*15*	175 (10.9)		
*16*	161 (10.0)		
*17*	166 (10.4)		
Education – no. (%)*		Education – no. (%)*	
*No current education*	8 (0.5)	*Low*	252 (14.1)
*Primary school*	12 (0.7)	*Intermediate*	1318 (73.9)
*Secondary school*	1406 (87.7)	*High*	214 (12.0)
- *preparing for vocational education*	552 (39.3)		
- *preparing for higher education*	809 (57.5)		
- *Combination of preparing for vocational an higher education*	45 (3.2)		
*Vocational education:*	129 (8.0)		
*Higher education:*	48 (0.7)		
*Total – no. (%)*	1603 (100)	*Total – no. (%)*	1714 (100)

* operationalization based upon: [8]

**Table 3:** Characteristics of healthcare professionals in this study

Characteristic healthcare professionals	
*Female – no. (%)*	329 (63.3)
*Age in years – range (mean)*	25–70 (46.6)
*Occupation in years – range (mean)*	1–46 (14.2)
*Work area description – no. (%)*	
*Rural*	114 (21.9)
*Urban*	221 (42.5)
*Semi-rural, semi-urban*	185 (35.6)
*Total no. (%)*	520 (100)

### Information needs of teenagers and parents

**Fig. 1:**
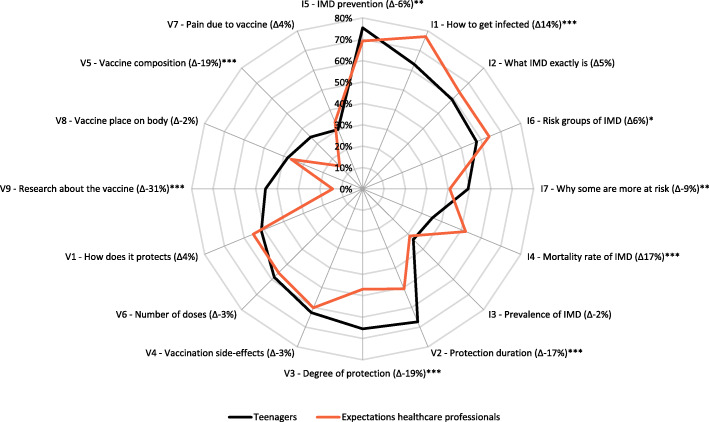
Percentages of teenagers who perceived information items about IMD (I1–7)^#^ and the menACWY vaccination (V1–9)^#^ as important, and percentages of healthcare professionals who expected that teenagers would perceive these information items as important. The information items are ranked clockwise (most important to least important as indicated by teenagers), separately for I items and V items. # The numbers of the information items in the Figure correspond to the information items in Table 1. ∆ Difference between the percentage of teenagers that indicated an item as important to them and the percentage of healthcare professionals that expected that teenagers would indicate this item as important to them. A positive difference indicates higher expected importance in healthcare professionals than indicated by teenagers, and a negative difference indicates a lower expected importance by healthcare professionals than indicated by teenagers. * Significant (*p* < 0.05) difference between the percentage of teenagers indicating an item as important/less important to them and the percentage of healthcare professionals expecting that parents would indicate this item as important/less important. ** Significant (*p* < 0.01) difference between the percentage of teenagers indicating an item as important/less important to them and the percentage of healthcare professionals expecting that parents would indicate this item as important/less important. *** Significant (*p* < 0.001) difference between the percentage of teenagers indicating an item as important/less important to them and the percentage of healthcare professionals expecting that parents would indicate this item as important/less important

**Fig. 2:**
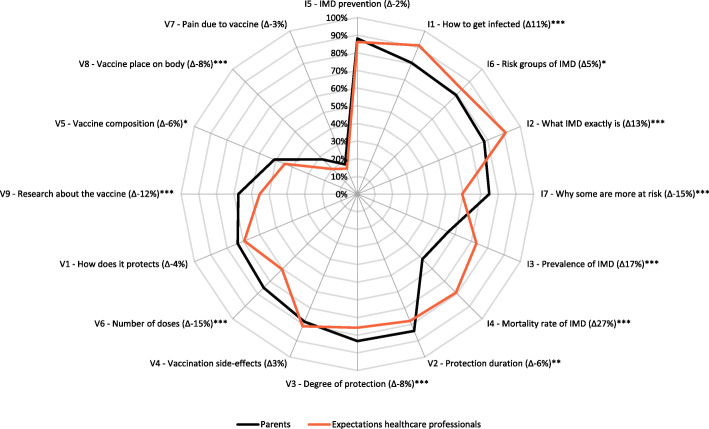
Percentages of parents who perceived information items about IMD (I1–7)^#^ and the menACWY vaccination (V1–9)^#^ as important, and percentages of healthcare professionals who expected that parents would perceive these information items as important. The information items are ranked clockwise (most important to least important as indicated by parents), separately for I items and V items. # The numbers of the information items in the Figure correspond to the information items in Table 1. ∆ Difference between the percentage of parents that indicated an item as important to them and the percentage of healthcare professionals that expected that parents would indicate this item as important to them. A positive difference indicates higher expected importance in healthcare professionals than indicated by parents, and a negative difference indicates a lower expected importance by healthcare professionals than indicated by parents. * Significant (*p* < 0.05) difference between the percentage of parents indicating an item as important/less important to them and the percentage of healthcare professionals expecting that parents would indicate this item as important/less important. ** Significant (*p* < 0.01) difference between the percentage of parents indicating an item as important/less important to them and the percentage of healthcare professionals expecting that parents would indicate this item as important/less important. *** Significant (*p* < 0.001) difference between the percentage of parents indicating an item as important/less important to them and the percentage of healthcare professionals expecting that parents would indicate this item as important/less important

The percentages of teenagers and parents that perceived IMD and menACWY information items important to them are shown in Figs. 1 and 2. Teenagers and parents generally prioritized the same IMD information as important information, but parents perceived all IMD information items (7/7) and most menACWY information items (7/9) as significantly (*p* < 0.001) more important than teenagers.

The IMD items that were most often indicated as important by both teenagers and parents were “how can you prevent IMD” (I5; 75% of the teenagers (*N* = 1208), 88% of the parents (*N* = 1572)) and “how can you get infected with IMD” (I1; 63% of the teenagers (*N* = 1011), 80% of the parents (*N* = 1435)). The vaccination information items that were most often indicated as important were “how long this vaccination protects against IMD” (V2; 67% of the teenagers (*N* = 1079), 84% of the parents (*N* = 1499)), “to what extent this vaccination protects against IMD” (V3; 66% of the teenagers (*N* = 1050), 83% of the parents (*N* = 1487)), and “what are the possible side-effects of this vaccination” (V4; 63% of the teenagers (*N* = 1005), 78% of the parents (*N* = 1398)).

### Healthcare professionals’ expectations of information needs among teenagers and parents

Figures 1 and 2 additionally show the differences between the information needs in teenagers and parents, respectively, and the expectations of these information needs in healthcare professionals. For several IMD and menACWY information items, healthcare professionals’ expectations were significantly (*p* < 0.05) different from the responses by teenagers and parents. In general, healthcare professionals somewhat overestimated parents’ and teenagers’ information needs about IMD and underestimated parents’ and teenagers’ information needs about the menACWY vaccination. The largest difference between healthcare professionals’ expectations and teenagers’ menACWY information needs was observed for the item: “the research that has been conducted on this vaccination” (V9); 14% of the healthcare professionals (*N* = 73) expected that teenagers would perceive this information as important and 45% of the teenagers (*N* = 729) indicated that this information was important to them. The largest difference between healthcare professionals expectations and parents’ responses to menACWY information items was observed for the item “how often you need to get vaccinated against IMD” (V6); 60% of the healthcare professionals (*N* = 314) expected that parents would perceive this information as important and 75% of the parents (*N* = 1340) indicated to perceive this information as important.

### Information needs among healthcare professionals

**Fig. 3:**
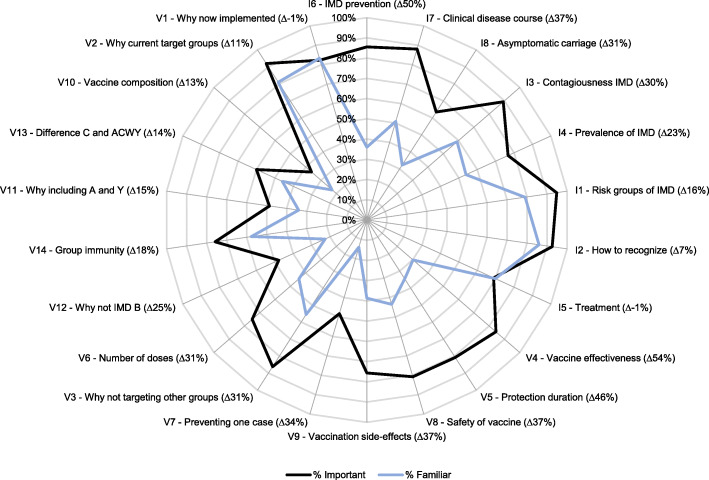
Healthcare professionals’ indicated importance of and familiarity with information about IMD (I1–8)^#^ and the menACWY vaccination (V1–14)^#^. The information items are ranked clockwise (largest to smallest differences between indicated importance and familiarity), separately for I items and V items. # The numbers of the information items in the Figure correspond to the information items in Table 1. ∆ Difference in the percentage of healthcare professionals indicating an item as important on the one hand, and the percentage of healthcare professionals indicating that item as familiar on the other (%important - %familiar)

Figure 3 shows the response percentages of perceived importance and familiarity with the IMD and menACWY information items in healthcare professionals. Healthcare professionals considered most of the IMD and menACWY information items more often as important (mean percentage 74%) than as familiar (mean percentage 49%). The largest differences between the indicated importance and familiarity in items were observed for: “how can you prevent IMD (besides vaccinations)” (I6; 86% important (*N* = 445), 36% familiar (*N* = 187)), “how effective the vaccine is” (V4; 84% important (*N* = 439), 30% familiar (*N* = 157)) and “how long the vaccine protects” (V5; 81% important (*N* = 420), 35% familiar (*N* = 180)).

## Discussion

Information concerning the mode of transmission and prevention of IMD, as well as information about the effectiveness and protection duration of the menACWY vaccine, were especially perceived as important by both teenagers and parents. Healthcare professionals’ expectations of the information needs of teenagers and parents were quite accurate but some important discrepancies were found. Furthermore, healthcare professionals were unfamiliar with various types of information which they did perceive as important in their communication with teenagers and parents. The largest knowledge gaps/information needs in healthcare professionals (i.e. high perceived importance of the information and low familiarity with the information) were found for information about the prevention of IMD, and the effectiveness and protection duration of the menACWY vaccine.

The observed difference between a number of teenagers’ and parents’ indicated information needs and healthcare professionals’ expectations of these information needs might indicate that healthcare professionals were not optimally prepared for questions from teenagers and parents. Overall, healthcare professionals seemed to somewhat overestimate teenagers’ and parents’ information needs about IMD and somewhat underestimate their information needs about the menACWY vaccination. Although most of these differences were rather small, and thus indicate that healthcare professionals’ expectations of teenagers and parents were fairly accurate, some differences were considerable. This was for example the case for information about the research that has been done on the menACWY vaccination. This information was perceived considerably more important by teenagers than was expected by healthcare professionals. Possibly more striking were the commonalities in the information needs among teenagers and parents concerning IMD and the menACWY vaccination and the information needs among healthcare professionals in their communication with teenagers and parents. Teenagers, parents and healthcare professionals all showed to have considerable information needs regarding IMD prevention, and vaccine effectiveness and protection duration.

The gaps in knowledge in healthcare professionals concerning specific disease and vaccine information as well as their underestimation of certain information needs about the menACWY vaccination might have caused problems during consultations with teenagers and parents. A literature study showed that negative experiences with vaccination consultations with healthcare professionals have been associated with vaccine refusal [9]. It has further been argued that when people do not feel sufficiently informed after a vaccination consult with a healthcare professional, they are likely to seek the missing information on the internet, which might lead them to vaccine skeptical websites [10]. Previous studies have also shown that if healthcare professionals do not have sufficient knowledge about a specific disease or vaccine or if they do not feel comfortable explaining the risks and benefits of the vaccine, that they are less likely to recommend the vaccination to their clients [4, 5].

Our study has some limitations. First, the survey response among healthcare professionals was sub-optimal. This was specifically the case for youth healthcare professionals. This limited response confines the generalizability of our results for this group of healthcare professionals. Second, we need to be cautious with the interpretation of the differences between teenagers’ and parents’ information needs and healthcare professionals’ expectations of these information needs. There is not an objective benchmark to assess the differences between the responses of teenagers/parents and the expectations of healthcare professionals as small or large. Third, our study might include an oversample of people with a specific interest in this topic, as participation to the surveys was voluntary.

In conclusion, our results suggest that healthcare professionals might not have been optimally equipped or prepared for questions from teenagers and their parents about IMD and the menACWY vaccination at the beginning of the vaccination campaign. This could indicate that the communication between public health organizations and healthcare professionals has not been optimal, which, in a previous study, was identified as one of the main barriers to physician delivered immunizations [11]. Future research might need to shed more light on communication about vaccinations between public health institutes and healthcare professionals, and on how this communication is perceived by both parties. We recommend public health institutes to timely assess and meet information needs about new vaccines in healthcare professionals with an important role in communication, to optimally equip them for, on their turn, meeting the information needs of their clients.

## Data Availability

The datasets used and/or analysed during the current study available from the corresponding author on reasonable request.

## Abbreviations

CFR: Case Fatality Rate
GP: General practitioner
IMD: Invasive Meningococcal Disease
NIP: National Immunization Program
RIVM: National Institute for Public Health and the Environment in the Netherlands (In Dutch: *Rijksinstituut voor Volksgezondheid en Milieu*)
